# Preliminary Feasibility Study of Benzo(a)Pyrene Oxidative Degradation by Fenton Treatment

**DOI:** 10.1155/2009/149034

**Published:** 2009-10-08

**Authors:** Vera Homem, Zélia Dias, Lúcia Santos, Arminda Alves

**Affiliations:** LEPÆ, Departamento de Engenharia Química, Faculdade de Engenharia da Universidade do Porto, Rua Dr. Roberto Frias, 4200-465 Porto, Portugal

## Abstract

Polycyclic aromatic hydrocarbons (PAHs) are considered priority compounds due to their toxic and carcinogenic nature. The concern about water contamination and the consequent human exposure has encouraged the development of new methods for PAHs removal. The purpose of this work was to study the feasibility of a degradation process of benzo(a)pyrene (BaP) in aqueous matrices by oxidation with Fenton reagent. A laboratory unit was designed to optimize the factors which may influence the process: pH (3.5 to 6.0), temperature (30 to 70°C), H_2_O_2_ (20 to 150 mg L^−1^), Fe^2+^ concentration (2.75 to 5.50 mg L^−1^), and the initial concentration of the pollutant (10 to 100 *μ*g L^−1^). The pH did not influence significantly the results in the range studied. An increase in temperature from 30 to 70°C improved the removal efficiency from 90% to 100%. The same effect was observed for ferrous ion concentrations from 2.75 to 5.50 mg L^−1^ (increase from 78% to 100% removal). The H_2_O_2_ concentration played a double role during the process: from 20 to 50 mg L^−1^ an increase in the removal efficiency was achieved, but for higher concentrations (>50 mg L^−1^) the degradation is lower. This study proved that the degradation of benzo(a)pyrene by Fenton's reagent is a viable process.

## 1. Introduction

In these last years, an increasing concern about monitoring water quality has been reflected in many studies. The amount of freshwater on Earth is limited and its quality constantly threatened. Hence there is a demand for the protection of water resources, in order to prevent their contamination by toxic compounds and pathogenic agents. Nowadays, the major concern is focused on organic pollutants such as polycyclic aromatic hydrocarbons (PAHs). 

PAHs are compounds with two or more fused aromatic rings, containing only carbon and hydrogen [1]. They may enter the environment by either natural or anthropogenic sources. The former includes volcanic eruptions and forest fires. However, the largest fraction is produced by the latter, namely, by incomplete combustion of fossil fuels, petrochemical processing, automobile exhausts, and tobacco smoke [2–4]. These compounds provoke adverse effects in the ecosystems, even at low concentrations (ng-*μ*g L^−1^). They are toxic and persistent, reveal bioaccumulation effects [5], and are endocrine disrupting as well as tumorigenic substances [6]. In addition, PAHs with four or more rings are carcinogenic and mutagenic as a result of their ability to suffer metabolic transformations [7]. 

The Water Framework Directive (2000/60/EC) outlined a strategy to combat water pollution and also demanded the establishment of a list of priority pollutants [8]. In the Decision 2455/2001/EC, thirty three substances or groups of substances have been selected to be monitored by the EU member states. Eight PAHs are included in that list: anthracene, fluoranthene, naphthalene, benzo(a)pyrene, benzo(b)fluoranthene, benzo(k)fluoranthene, benzo(g,h,i)perylene, and indeno(1,2,3-cd)pyrene) [9]. Sixteen PAHs are also listed by the Environmental Protection Agency (EPA) [10] due to their toxicity—the eight mentioned above and acenaphtalene, acenaphthylene, fluorene, phenanthrene, benzo(a)anthracene, chrysene, pyrene, and dibenzo(a,h)anthracene.

Benzo(a)pyrene (BaP), one of the most toxic PAHs, is usually selected as an indicator of the presence of other compounds belonging to that group [11]. It has been detected in a diversity of aqueous matrices such as surface waters, seawaters, groundwater, drinking water, as well as in sediments [12]. Maximum limits of 0.010 *μ*gL^−1^ for drinking waters [13] and 0.1 *μ*g L^−1^ for water surfaces [14] have been set for BaP.

Given the risks posed by these compounds to public health, several methodologies for the decontamination of environmental matrices have been developed. Some authors suggest removal through volatilization, oxidation, adsorption to soil particles, and biodegradation [4]. As a result of the low biodegradability of PAHs, advanced oxidation processes (AOPs) have been studied as treatment methods [15, 16]. They should be applied as an alternative or a complement to the conventional treatments. Among the AOPs, the Fenton method is one of the most promising treatments, due to its high performance, technological simplicity, and moderate cost [16–19].

There are few studies about the degradation of PAHs via Fenton oxidative process in aqueous matrices. Beltrán et al. [20] investigated the aqueous oxidation of three PAHs (fluorene, phenanthrene, and acenaphthene), determining the influence of the main process variables and the products resulting from oxidation. The authors achieved a degradation of 80%, 97%, and 73%, respectively, (10 minutes) with initial concentrations of 0.9, 0.4, and 2 mg L^−1^, which are above the values commonly found in the environment. Nadarajah et al. [4] studied the potential use of Fenton's reagent as a pretreatment process to improve microbial treatment of anthracene and BaP in an aqueous system. The studies were conducted with an initial concentration of 100 mg L^−1^ for BaP. The application of Fenton's reagent, biodegradation, and the combination of both were tested. In the first case, about 15% of BaP removal was reached after 48 hours. In the biotreatment, the removal percentage was higher, about 30% in seven days. However, using the combination of both approaches, 80% of the pollutant was removed. It is important to point out that concentrations used in this study were also far from those found in naturally contaminated matrices. Besides that and given the level of degradation reached, the reaction time was too long. Flotron et al. [16] also tested the use of Fenton's reagent to degrade three PAHs (fluoranthene, benzo(b)fluoranthene, and BaP) in sewage sludges at an initial concentration of 80 *μ*g L^−1^. They concluded that BaP was the most easily degraded PAH, through hydroxyl radical oxidation, resulting in a removal of 85% after three hours. As mentioned in the previous case, the reaction time was long.

There are several studies describing PAHs degradation in other matrices such as soils and sediments [16, 21–25]. Lundstedt et al. [26] reviewed the sources, fate, and toxic hazards of PAH contaminated sites and mentioned several by-products formed during oxidation reactions. This topic is currently of major concern.

The present work pretends to evaluate the feasibility of BaP degradation (at *μ*g L^−1^ levels) in water matrices applying Fenton's reagent. The effect of variables that influence the Fenton degradation (temperature, initial concentrations of ferrous salt and hydrogen peroxide, and initial concentration of the analyte) was determined. 

## 2. Materials and Methods

### 2.1. Reagents and Standards

A commercial solution of benzo(a)pyrene (1000 *μ*g mL^−1^ in acetone) was obtained from Supelco (Bellefonte, PA, USA). From it, a 10 mg L^−1^ stock solution in ethanol was prepared as a base to calibration standards with concentrations of 1, 10, 40, 60, and 100 *μ*g L^−1^ prepared in deionised water. The ethanol absolute (p.a.) was purchased to Panreac (Barcelona, Spain). From the stock solution, two control standards (10 and 100 *μ*g L^−1^) were prepared weekly. 

Hydrogen peroxide in stable form (30% Perhydrol, p.a.) and iron (II) sulfate heptahydrate were purchased from Merck (Darmstadt, Germany). The pH of the PAH solutions was adjusted with H_2_SO_4_ 1 M (Merck). Acetonitrile HPLC grade was obtained from BDH Prolabo (Poole, UK).

### 2.2. Equipment

#### 2.2.1. Experimental Procedure

**Figure 1:**
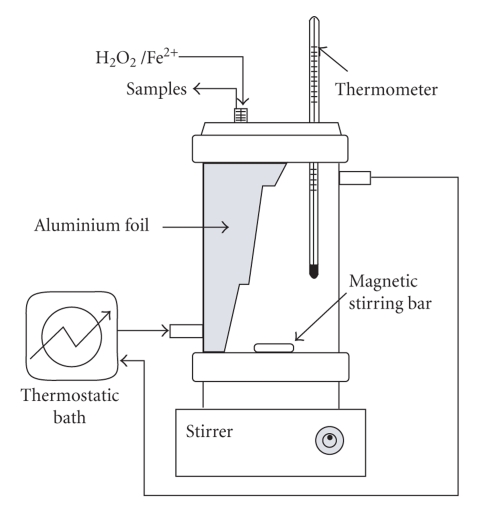
Scheme of the experimental device.

The experiments were conducted in a 250 mL jacketed thermostatic batch reactor (inner diameter: 7.5 cm, height: 11.5 cm). The outside of the reactor was covered with aluminium foil to protect from light, and an inlet for temperature measuring was placed on the top of the reactor. Homogeneous mixing was provided using a magnetic stirring bar and the temperature was kept constant with a thermostatic bath (Figure 1).

 In each experiment, 100 mL of BaP solution at the desired initial concentration were inserted in the reactor. An aliquot was withdrawn for further analysis. After that, the pH was adjusted with a sulphuric acid solution and another aliquot was collected. Then, the required amount of iron (II) salt was added. When the salt was totally dissolved, a certain quantity of H_2_O_2_ solution was introduced, in order to start the reaction. Table 1 shows the conditions applied to each experiment performed. Aliquots were taken from the reactor at selected time intervals and immediately analyzed. The arrest of Fenton's reaction was achieved with the addition of some drops of concentrated sulphuric acid in order to decrease the pH to less than 1.0 [27]. The option for sulphuric acid instead of sodium sulphite may be discussed, but it is acceptable to consider that the reaction rate is sufficiently decreased in order to allow the subsequent analysis.

#### 2.2.2. Analytical Method

HPLC analyses were performed with a Merck Hitachi LaChrom Elite system (Darmstadt, Germany) equipped with an L-2130 pump, L-2200 autosampler, and a L-2480 fluorescence detector. Data were acquired and processed by EZChrom Elite software from Agilent (Santa Clara, CA, USA). For chromatographic separation, a reversed-phase RP-18 endcapped Purospher STAR (250 mm × 4 mm, particle size 5 *μ*m) was used, combined with a guard column (4 mm × 4 mm i.d.) also Purospher STAR, at room temperature. The mobile phase consisted of acetonitrile (90%) and water (10%) running in isocratic conditions at a flow rate of 1.2 mL min^−1^. The injection volume was 50 *μ*L, and the excitation and emission wavelengths were 297 nm and 405 nm, respectively. Total run time was 15 minutes, and quantification was performed by external standard method.

## 3. Results and Discussion

### 3.1. Validation of the Analytical Method

The method linearity was verified in the 1 to 100 *μ*g L^−1^ range (five calibration points), obtaining a coefficient of determination (R^2^) of 0.9993 and a limit of detection (LOD) of 2.4 *μ*g L^−1^, calculated from the calibration curve. Other validation parameters were evaluated in the linearity range: precision varied between 3.5% and 13.9% and accuracy from 71.3% to 85.3%. The uncertainty associated to the analytical method was calculated according to the EURACHEM/CITAC guide [28]. The global uncertainty values obtained ranged from 2.4% to 59.1%. 

### 3.2. Oxidation Studies

As mentioned above, Fenton's reagent is a strong oxidant mixture consisting of hydrogen peroxide and iron (II) salt that acts as a catalyst. In this process, the hydroxyl radicals are formed in situ and depend on several factors such as pH, temperature, and the initial concentrations of hydrogen peroxide, ferrous ion, and BaP, whose effects were investigated in this work.

The standards of BaP were prepared in water (neutral pH). However, the Fenton's reaction occurs in acidic conditions. For that reason, it was necessary to compare the fluorescence response before and after the addition of sulphuric acid, and it was verified that such responses remained practically unchanged. Another central issue is the arrest of Fenton's reaction, which is usually done using sodium sulphite. Nevertheless, in this work the stop was achieved with the addition of some drops of concentrated sulphuric acid in order to decrease the pH to less than 1.0. At pH < 1 an inhibition in the production of hydroxyl radicals occurs, due to H^+^ ions scavenging. Therefore, the amount of OH^•^ is strongly reduced and, consequently, the reaction rate is very slow. This methodology is valid once the analyses were performed in a short time interval after the addition of acid to the aliquots; otherwise slight variation of the concentration may occur.

**Effect of pH**

**Figure 2:**
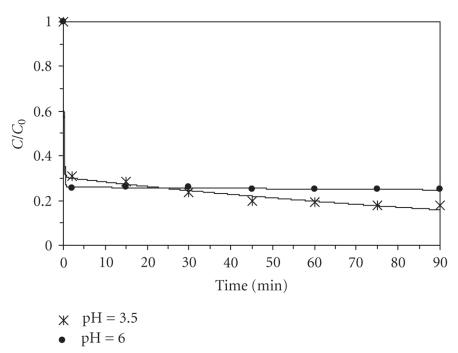
Effect of pH on BaP oxidation with Fenton's reagent (10 *μ*g L^−1^BaP, 40°C, 5.5 mg L^−1^Fe^2+^, 200 mg L^−1^H_2_O_2_).

The Fenton's reaction is pH dependent, because this value affects the hydroxyl radicals generation and, consequently, the oxidation efficiency. For this degradation process, the optimal pH range mentioned in literature is 3 to 6. Therefore, in this work the 3.5 and 6.0 pH values were studied and the results are shown in Figure 2. It can be observed that the removal efficiency was not significantly changed with the pH increase from 3.5 to 6. In subsequent experiments, pH = 3.5 was used in order to compare the results with those presented in most of the previous studies reported in literature.

**Effect of Temperature**

**Figure 3:**
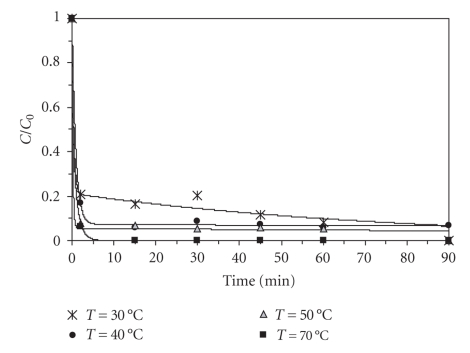
Effect of temperature on BaP degradation (10 *μ*g L^−1^BaP, pH = 3.5, 5.5 mg L^−1^Fe^2+^, 200 mg L^−1^H_2_O_2_).

Experiments were conducted under the same conditions at four different temperatures between 30 and 70°C to investigate the effect of temperature on the degradation kinetics of aqueous BaP solutions. The results are illustrated in Figure 3. An enhancement in the rate and even in the extent of degradation reaction was observed with the temperature increase. Despite this, at higher temperatures the thermal decomposition of hydrogen peroxide may be accelerated, resulting in a decrease of the concentration of hydroxyl radicals, with consequent reduction in the reaction extent. On the other hand, there was practically no difference between the experiments carried out at 40 and 50°C (removal of 90%). The economic aspect is often a limiting factor; thus the best option would be working at 40°C.

**Effect of Hydrogen Peroxide Concentration**

**Figure 4:**
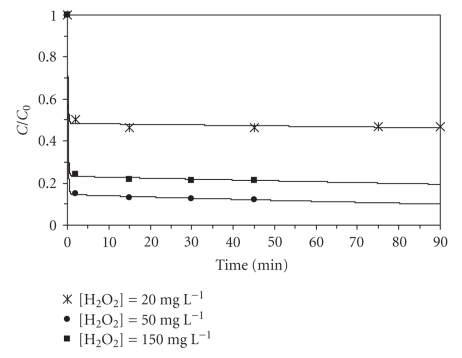
Influence of hydrogen peroxide concentration on BaP degradation (10 *μ*g L^−1^BaP, pH = 3.5, 40°C, 3.75 mg L^−1^Fe^2+^).

Experiments were performed to determine the effect of hydrogen peroxide concentration on the process (Figure 4). In all experiments, it was observed that the maximum degradation of BaP concentration was reached after two minutes. On the other hand, from Figure 4 it can also be seen that the increase of hydrogen peroxide concentration from 20 to 50 mg L^−1^ yields rising removal efficiencies. However, higher concentrations lead to lower degradation rates. The recombination of hydroxyl radicals and the reaction between them and hydrogen peroxide may explain this fact.

**Effect of Ferrous Ion Concentration**

**Figure 5:**
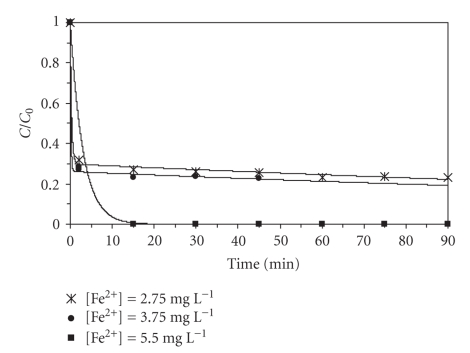
Effect of the initial concentration of ferrous ion on BaP degradation (10 *μ*g L^−1^BaP, pH = 3.5, 40°C, 100 mg L^−1^H_2_O_2_).

Experiments were conducted in order to investigate the effect of ferrous ion concentration (catalytic agent) on the process. Figure 5 shows the relationship between the degradation extent and the initial concentration of ferrous ion. Comparing the results, it was noticed that there is an increasing degradation with the Fe^2+^ concentration (70% to 100% removal), although no significant differences were found between 2.75 and 3.75 mg L^−1^.

In 1996, Béltran et al. [20] established the influence of Fe^2+^ in the fluorene degradation (0.9 mg L^−1^). The initial ferrous ion concentration ranged between 0.6 and 11 mg L^−1^. They showed that augmenting this concentration improved the degradation (40% to 100% removal) as well as the reaction rate. The same conclusion was obtained in this study.

The homogeneous Fenton process has the disadvantage of commonly using high concentrations of ferrous ion (50 to 80 mg L^−1^), which is beyond the legal limit of 2 mg L^−1^ for treated water to be released directly into the environment [29]. In this work, a maximum ferrous ion concentration of 5.50 mg L^−1^ was applied. Therefore, a dilution of the treated effluent may be sufficient to achieve legal conformity.

**Effect of Initial BaP Concentration**

**Figure 6:**
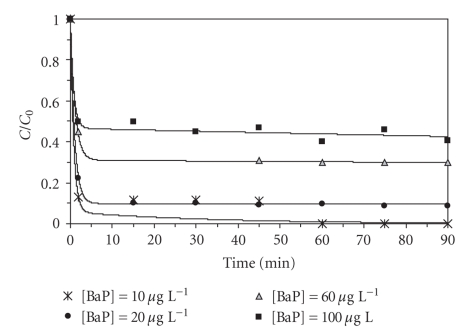
Effect of BaP initial concentration (pH = 3.5, 40°C, 50 mg L^−1^ H_2_O_2_, 3.75 mg L^−1^ Fe^2+^).

Thinking about a possible application of this method to naturally contaminated samples, it is important to study the dependence of the degradation efficiency on the initial concentration of the analyte. In wastewater treatment plants, the analyte concentration present in the effluent is usually unknown. Therefore, it is essential to determine the maximum amount of pollutant that would be degraded with a fixed reagent concentration. As seen in Figure 6, the reaction occurs quickly in the first 10 minutes and then stabilizes at the maximum degradation value, for all cases studied. After a period of 90 minutes a removal of 100%, 90%, 70%, and 57% was, respectively, achieved with 10, 20, 60, and 100 *μ*g L^−1^BaP, plus 3.75 mg L^−1^Fe^2+^ and 50 mg L^−1^H_2_O_2_.

There are two main problems related to the micropollutants degradation by Fenton's reagent: sludge production and generation of by-products. Normally the total mineralization of the compounds does not occur, and the process generates metabolites equally or even more toxic than the original compounds. To check the possibility of applying another type of treatment (e.g., biodegradation) or discharge the effluent, the identification of these metabolites becomes an important issue, to be investigated in a subsequent study.

## 4. Conclusions

The main conclusion of the present work is that Fenton's reagent is an appropriate method for the total degradation of benzo(a)pyrene in water matrices, providing that the ferrous ion and hydrogen peroxide are present in suitable concentrations. These parameters as well as temperature are important variables for the process. It was shown that an increase in temperature from 30 to 70°C led to an increase in the removal efficiency from 90% to 100%. The same effect was verified with the increase of the ferrous ion concentration from 2.75 to 5.50 mg L^−1^ (removals from 78% to 100%). The hydrogen peroxide was the only reagent with a double role during the oxidation: despite the degradation of BaP increased with the H_2_O_2_ concentration, at high concentrations of oxidant the removal was reduced. With an initial concentration of 50 mg L^−1^, 90% removal was achieved while with 150 mg L^−1^ only 80% BaP was eliminated. 

Future work will consider a scale-up optimization as well as the identification of the reaction by-products, if they appear.

## Figures and Tables

**Table 1:** Experimental conditions used in Fenton's reaction.

Run no.	pH	T(°C)	[BaP]_0_ (*μ*g L^−1^)	[H_2_O_2_]_0_ (mg L^−1^)	[Fe^2+^]_0_ (mg L^−1^)
1	3.5	40	10	200	5.50
2	6.0	40	10	200	5.50
3	3.5	30	10	200	5.50
4	3.5	50	10	200	5.50
5	3.5	70	10	200	5.50
6	3.5	40	10	20	3.75
7	3.5	40	10	50	3.75
8	3.5	40	10	150	3.75
9	3.5	40	10	100	2.75
10	3.5	40	10	100	5.50
11	3.5	40	10	100	3.75
12	3.5	40	20	50	3.75
13	3.5	40	60	50	3.75
14	3.5	40	100	50	3.75
